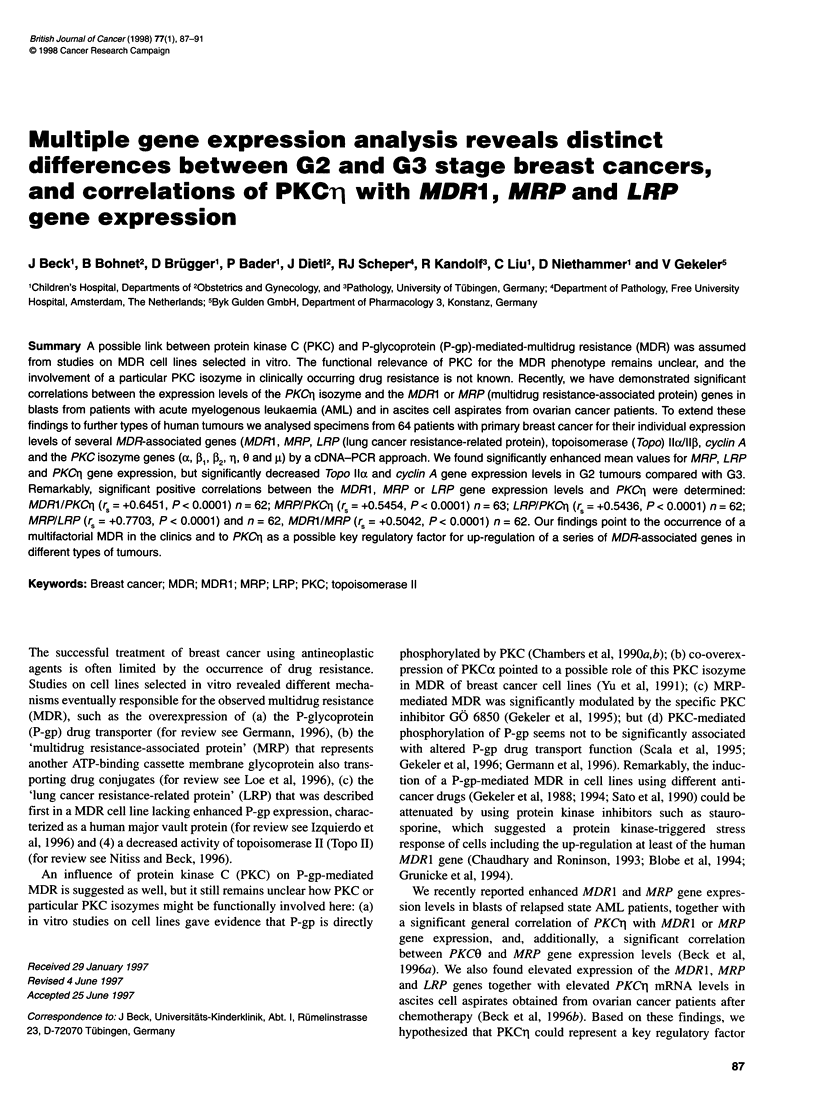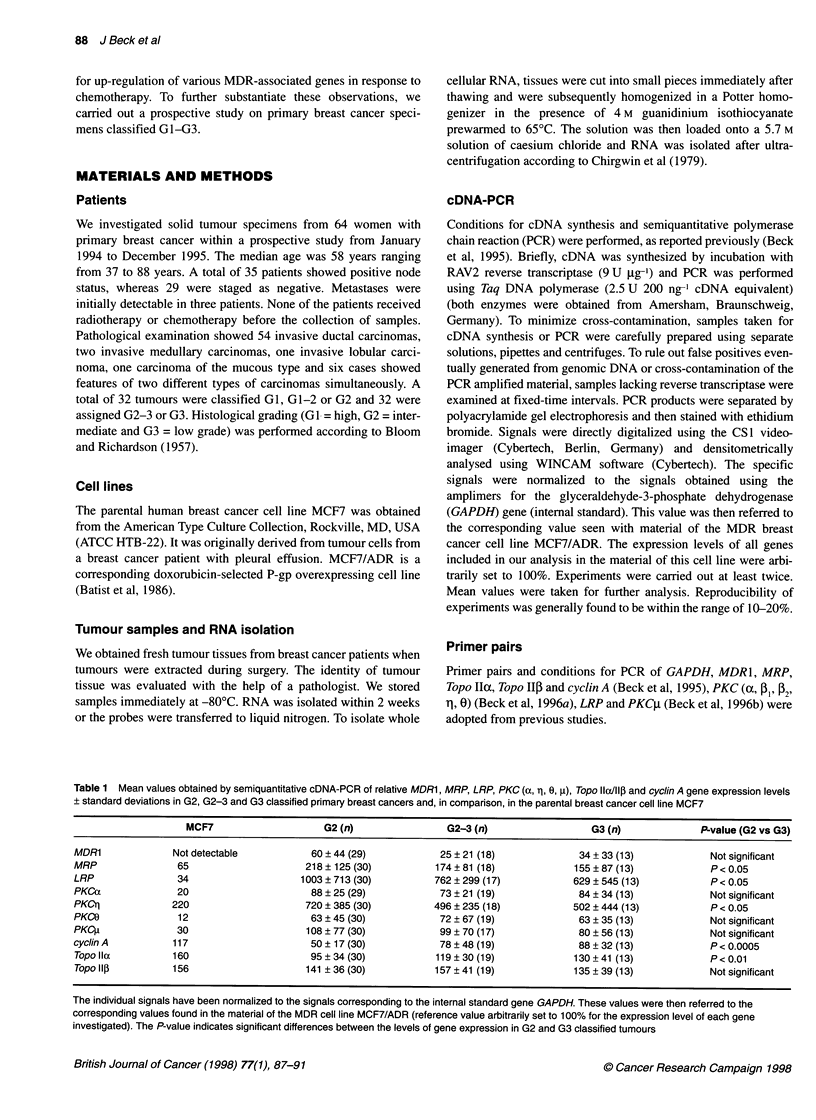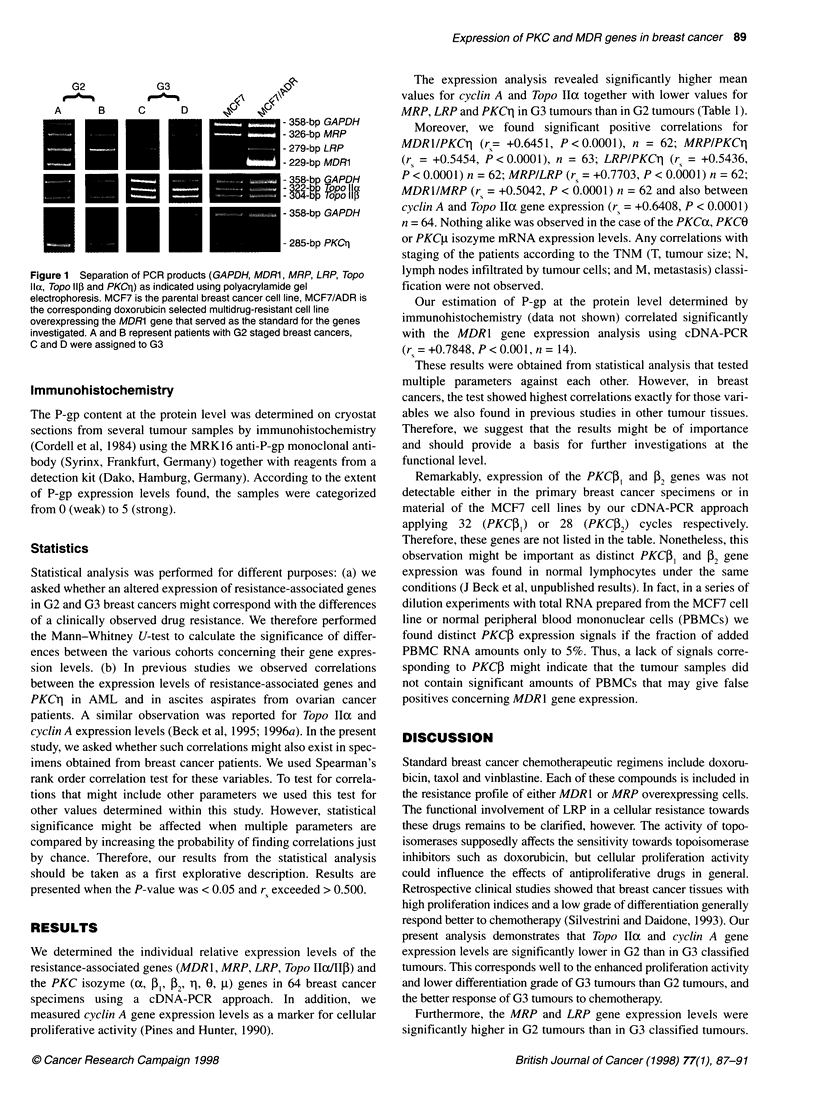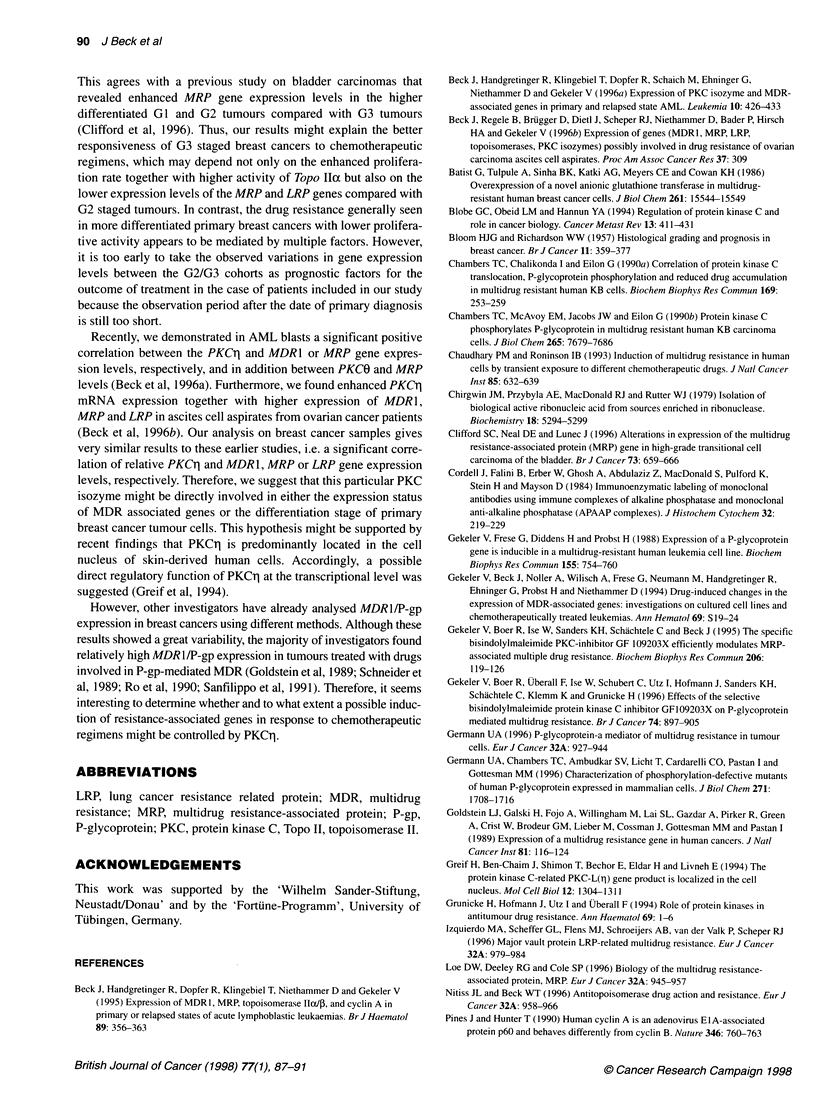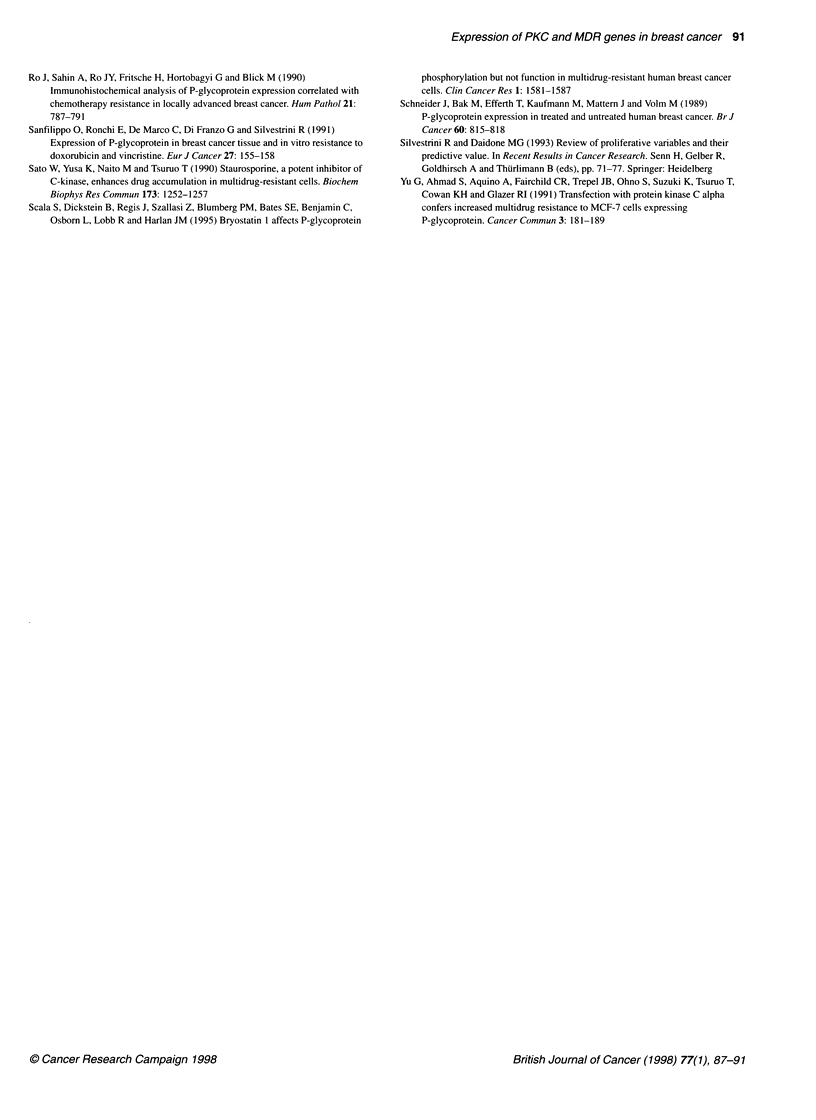# Multiple gene expression analysis reveals distinct differences between G2 and G3 stage breast cancers, and correlations of PKC eta with MDR1, MRP and LRP gene expression.

**DOI:** 10.1038/bjc.1998.13

**Published:** 1998

**Authors:** J. Beck, B. Bohnet, D. BrÃ¼gger, P. Bader, J. Dietl, R. J. Scheper, R. Kandolf, C. Liu, D. Niethammer, V. Gekeler

**Affiliations:** Children's Hospital, TÃ¼bingen, Germany.

## Abstract

**Images:**


British Journal of Cancer (1998) 77(1), 87–91
© 1998 Cancer Research Campaign

### Multiple gene expression analysis reveals distinct differences between G2 and G3 stage breast cancers, and correlations of PKCη with *MDR*1, *MRP* and *LRP* gene expression

**J Beck[1], B Bohnet[2], D Brügger[1], P Bader[1], J Dietl[2], RJ Scheper[4], R Kandolf[3], C Liu[1], D Niethammer[1] and V Gekeler[5]**

[1]Children's Hospital, Departments of [2]Obstetrics and Gynecology, and [3]Pathology, University of Tübingen, Germany; [4]Department of Pathology, Free University Hospital, Amsterdam, The Netherlands; [5]Byk Gulden GmbH, Department of Pharmacology 3, Konstanz, Germany

**Summary** A possible link between protein kinase C (PKC) and P-glycoprotein (P-gp)-mediated-multidrug resistance (MDR) was assumed from studies on MDR cell lines selected in vitro. The functional relevance of PKC for the MDR phenotype remains unclear, and the involvement of a particular PKC isozyme in clinically occurring drug resistance is not known. Recently, we have demonstrated significant correlations between the expression levels of the *PKC*η isozyme and the *MDR*1 or *MRP* (multidrug resistance-associated protein) genes in blasts from patients with acute myelogenous leukaemia (AML) and in ascites cell aspirates from ovarian cancer patients. To extend these findings to further types of human tumours we analysed specimens from 64 patients with primary breast cancer for their individual expression levels of several *MDR*-associated genes (*MDR*1, *MRP*, *LRP* (lung cancer resistance-related protein), topoisomerase (*Topo*) IIα/IIβ, *cyclin A* and the *PKC* isozyme genes (α, $\beta_1$, $\beta_2$, η, θ and μ) by a cDNA–PCR approach. We found significantly enhanced mean values for *MRP*, *LRP* and *PKC*η gene expression, but significantly decreased *Topo* IIα and *cyclin A* gene expression levels in G2 tumours compared with G3. Remarkably, significant positive correlations between the *MDR*1, *MRP* or *LRP* gene expression levels and *PKC*η were determined: *MDR*1/*PKC*η ($r_s$ = +0.6451, $P < 0.0001$) $n = 62$; *MRP*/*PKC*η ($r_s$ = +0.5454, $P < 0.0001$) $n = 63$; *LRP*/*PKC*η ($r_s$ = +0.5436, $P < 0.0001$) $n = 62$; *MRP*/*LRP* ($r_s$ = +0.7703, $P < 0.0001$) and $n = 62$, *MDR*1/*MRP* ($r_s$ = +0.5042, $P < 0.0001$) $n = 62$. Our findings point to the occurrence of a multifactorial MDR in the clinics and to *PKC*η as a possible key regulatory factor for up-regulation of a series of *MDR*-associated genes in different types of tumours.

**Keywords:** Breast cancer; MDR; MDR1; MRP; LRP; PKC; topoisomerase II

The successful treatment of breast cancer using antineoplastic agents is often limited by the occurrence of drug resistance. Studies on cell lines selected in vitro revealed different mechanisms eventually responsible for the observed multidrug resistance (MDR), such as the overexpression of (a) the P-glycoprotein (P-gp) drug transporter (for review see Germann, 1996), (b) the 'multidrug resistance-associated protein' (MRP) that represents another ATP-binding cassette membrane glycoprotein also transporting drug conjugates (for review see Loe et al, 1996), (c) the 'lung cancer resistance-related protein' (LRP) that was described first in a MDR cell line lacking enhanced P-gp expression, characterized as a human major vault protein (for review see Izquierdo et al, 1996) and (4) a decreased activity of topoisomerase II (Topo II) (for review see Nitiss and Beck, 1996).

An influence of protein kinase C (PKC) on P-gp-mediated MDR is suggested as well, but it still remains unclear how PKC or particular PKC isozymes might be functionally involved here: (a) in vitro studies on cell lines gave evidence that P-gp is directly phosphorylated by PKC (Chambers et al, 1990*a,b*); (b) co-overexpression of PKCα pointed to a possible role of this PKC isozyme in MDR of breast cancer cell lines (Yu et al, 1991); (c) MRP-mediated MDR was significantly modulated by the specific PKC inhibitor GÖ 6850 (Gekeler et al, 1995); but (d) PKC-mediated phosphorylation of P-gp seems not to be significantly associated with altered P-gp drug transport function (Scala et al, 1995; Gekeler et al, 1996; Germann et al, 1996). Remarkably, the induction of a P-gp-mediated MDR in cell lines using different anticancer drugs (Gekeler et al, 1988; 1994; Sato et al, 1990) could be attenuated by using protein kinase inhibitors such as staurosporine, which suggested a protein kinase-triggered stress response of cells including the up-regulation at least of the human *MDR*1 gene (Chaudhary and Roninson, 1993; Blobe et al, 1994; Grunicke et al, 1994).

We recently reported enhanced *MDR*1 and *MRP* gene expression levels in blasts of relapsed state AML patients, together with a significant general correlation of *PKC*η with *MDR*1 or *MRP* gene expression, and, additionally, a significant correlation between *PKC*θ and *MRP* gene expression levels (Beck et al, 1996*a*). We also found elevated expression of the *MDR*1, *MRP* and *LRP* genes together with elevated *PKC*η mRNA levels in ascites cell aspirates obtained from ovarian cancer patients after chemotherapy (Beck et al, 1996*b*). Based on these findings, we hypothesized that PKCη could represent a key regulatory factor

*Received 29 January 1997*
*Revised 4 June 1997*
*Accepted 25 June 1997*

*Correspondence to:* J Beck, Universitäts-Kinderklinik, Abt. I, Rümelinstrasse 23, D-72070 Tübingen, Germany for up-regulation of various MDR-associated genes in response to chemotherapy. To further substantiate these observations, we carried out a prospective study on primary breast cancer specimens classified G1–G3.

### MATERIALS AND METHODS

### Patients

We investigated solid tumour specimens from 64 women with primary breast cancer within a prospective study from January 1994 to December 1995. The median age was 58 years ranging from 37 to 88 years. A total of 35 patients showed positive node status, whereas 29 were staged as negative. Metastases were initially detectable in three patients. None of the patients received radiotherapy or chemotherapy before the collection of samples. Pathological examination showed 54 invasive ductal carcinomas, two invasive medullary carcinomas, one invasive lobular carcinoma, one carcinoma of the mucous type and six cases showed features of two different types of carcinomas simultaneously. A total of 32 tumours were classified G1, G1–2 or G2 and 32 were assigned G2–3 or G3. Histological grading (G1 = high, G2 = intermediate and G3 = low grade) was performed according to Bloom and Richardson (1957).

### Cell lines

The parental human breast cancer cell line MCF7 was obtained from the American Type Culture Collection, Rockville, MD, USA (ATCC HTB-22). It was originally derived from tumour cells from a breast cancer patient with pleural effusion. MCF7/ADR is a corresponding doxorubicin-selected P-gp overexpressing cell line (Batist et al, 1986).

### Tumour samples and RNA isolation

We obtained fresh tumour tissues from breast cancer patients when tumours were extracted during surgery. The identity of tumour tissue was evaluated with the help of a pathologist. We stored samples immediately at –80°C. RNA was isolated within 2 weeks or the probes were transferred to liquid nitrogen. To isolate whole cellular RNA, tissues were cut into small pieces immediately after thawing and were subsequently homogenized in a Potter homogenizer in the presence of 4 M guanidinium isothiocyanate prewarmed to 65°C. The solution was then loaded onto a 5.7 M solution of caesium chloride and RNA was isolated after ultracentrifugation according to Chirgwin et al (1979).

### cDNA-PCR

Conditions for cDNA synthesis and semiquantitative polymerase chain reaction (PCR) were performed, as reported previously (Beck et al, 1995). Briefly, cDNA was synthesized by incubation with RAV2 reverse transcriptase (9 U $\mu g^{-1}$) and PCR was performed using *Taq* DNA polymerase (2.5 U 200 $ng^{-1}$ cDNA equivalent) (both enzymes were obtained from Amersham, Braunschweig, Germany). To minimize cross-contamination, samples taken for cDNA synthesis or PCR were carefully prepared using separate solutions, pipettes and centrifuges. To rule out false positives eventually generated from genomic DNA or cross-contamination of the PCR amplified material, samples lacking reverse transcriptase were examined at fixed-time intervals. PCR products were separated by polyacrylamide gel electrophoresis and then stained with ethidium bromide. Signals were directly digitalized using the CS1 videoimager (Cybertech, Berlin, Germany) and densitometrically analysed using WINCAM software (Cybertech). The specific signals were normalized to the signals obtained using the amplimers for the glyceraldehyde-3-phosphate dehydrogenase (*GAPDH*) gene (internal standard). This value was then referred to the corresponding value seen with material of the MDR breast cancer cell line MCF7/ADR. The expression levels of all genes included in our analysis in the material of this cell line were arbitrarily set to 100%. Experiments were carried out at least twice. Mean values were taken for further analysis. Reproducibility of experiments was generally found to be within the range of 10–20%.

### Primer pairs

Primer pairs and conditions for PCR of *GAPDH*, *MDR*1, *MRP*, *Topo* IIα, *Topo* IIβ and *cyclin A* (Beck et al, 1995), *PKC* (α, $\beta_1$, $\beta_2$, η, θ) (Beck et al, 1996*a*), *LRP* and *PKC*μ (Beck et al, 1996b) were adopted from previous studies.

**Table 1** Mean values obtained by semiquantitative cDNA-PCR of relative *MDR*1, *MRP*, *LRP*, *PKC* (α, η, θ, μ), *Topo* IIα/IIβ and *cyclin A* gene expression levels ± standard deviations in G2, G2–3 and G3 classified primary breast cancers and, in comparison, in the parental breast cancer cell line MCF7

| | MCF7 | G2 (*n*) | G2–3 (*n*) | G3 (*n*) | *P*-value (G2 vs G3) |
|---|---|---|---|---|---|
| *MDR*1 | Not detectable | 60 ± 44 (29) | 25 ± 21 (18) | 34 ± 33 (13) | Not significant |
| *MRP* | 65 | 218 ± 125 (30) | 174 ± 81 (18) | 155 ± 87 (13) | $P < 0.05$ |
| *LRP* | 34 | 1003 ± 713 (30) | 762 ± 299 (17) | 629 ± 545 (13) | $P < 0.05$ |
| *PKC*α | 20 | 88 ± 25 (29) | 73 ± 21 (19) | 84 ± 34 (13) | Not significant |
| *PKC*η | 220 | 720 ± 385 (30) | 496 ± 235 (18) | 502 ± 444 (13) | $P < 0.05$ |
| *PKC*θ | 12 | 63 ± 45 (30) | 72 ± 67 (19) | 63 ± 35 (13) | Not significant |
| *PKC*μ | 30 | 108 ± 77 (30) | 99 ± 70 (17) | 80 ± 56 (13) | Not significant |
| *cyclin A* | 117 | 50 ± 17 (30) | 78 ± 48 (19) | 88 ± 32 (13) | $P < 0.0005$ |
| *Topo* IIα | 160 | 95 ± 34 (30) | 119 ± 30 (19) | 130 ± 41 (13) | $P < 0.01$ |
| *Topo* IIβ | 156 | 141 ± 36 (30) | 157 ± 41 (19) | 135 ± 39 (13) | Not significant |

The individual signals have been normalized to the signals corresponding to the internal standard gene *GAPDH*. These values were then referred to the corresponding values found in the material of the MDR cell line MCF7/ADR (reference value arbitrarily set to 100% for the expression level of each gene investigated). The *P*-value indicates significant differences between the levels of gene expression in G2 and G3 classified tumours

© Cancer Research Campaign 1998

**Figure 1** Separation of PCR products (*GAPDH, MDR*1, *MRP, LRP, Topo* IIα, *Topo* IIβ and *PKC*η) as indicated using polyacrylamide gel electrophoresis. MCF7 is the parental breast cancer cell line, MCF7/ADR is the corresponding doxorubicin selected multidrug-resistant cell line overexpressing the *MDR*1 gene that served as the standard for the genes investigated. A and B represent patients with G2 staged breast cancers, C and D were assigned to G3

### Immunohistochemistry

The P-gp content at the protein level was determined on cryostat sections from several tumour samples by immunohistochemistry (Cordell et al, 1984) using the MRK16 anti-P-gp monoclonal antibody (Syrinx, Frankfurt, Germany) together with reagents from a detection kit (Dako, Hamburg, Germany). According to the extent of P-gp expression levels found, the samples were categorized from 0 (weak) to 5 (strong).

### Statistics

Statistical analysis was performed for different purposes: (a) we asked whether an altered expression of resistance-associated genes in G2 and G3 breast cancers might correspond with the differences of a clinically observed drug resistance. We therefore performed the Mann–Whitney *U*-test to calculate the significance of differences between the various cohorts concerning their gene expression levels. (b) In previous studies we observed correlations between the expression levels of resistance-associated genes and *PKC*η in AML and in ascites aspirates from ovarian cancer patients. A similar observation was reported for *Topo* IIα and *cyclin A* expression levels (Beck et al, 1995; 1996*a*). In the present study, we asked whether such correlations might also exist in specimens obtained from breast cancer patients. We used Spearman's rank order correlation test for these variables. To test for correlations that might include other parameters we used this test for other values determined within this study. However, statistical significance might be affected when multiple parameters are compared by increasing the probability of finding correlations just by chance. Therefore, our results from the statistical analysis should be taken as a first explorative description. Results are presented when the *P*-value was < 0.05 and $r_s$ exceeded > 0.500.

### RESULTS

We determined the individual relative expression levels of the resistance-associated genes (*MDR*1, *MRP, LRP, Topo* IIα/IIβ) and the *PKC* isozyme (α, $\beta_1$, $\beta_2$, η, θ, μ) genes in 64 breast cancer specimens using a cDNA-PCR approach. In addition, we measured *cyclin A* gene expression levels as a marker for cellular proliferative activity (Pines and Hunter, 1990).

The expression analysis revealed significantly higher mean values for *cyclin A* and *Topo* IIα together with lower values for *MRP, LRP* and *PKC*η in G3 tumours than in G2 tumours (Table 1).

Moreover, we found significant positive correlations for *MDR*1/*PKC*η ($r_s$= +0.6451, $P < 0.0001$), $n$ = 62; *MRP*/*PKC*η ($r_s$ = +0.5454, $P < 0.0001$), $n$ = 63; *LRP*/*PKC*η ($r_s$ = +0.5436, $P < 0.0001$) $n$ = 62; *MRP*/*LRP* ($r_s$ = +0.7703, $P < 0.0001$) $n$ = 62; *MDR*1/*MRP* ($r_s$ = +0.5042, $P < 0.0001$) $n$ = 62 and also between *cyclin A* and *Topo* IIα gene expression ($r_s$ = +0.6408, $P < 0.0001$) $n$ = 64. Nothing alike was observed in the case of the *PKC*α, *PKC*θ or *PKC*μ isozyme mRNA expression levels. Any correlations with staging of the patients according to the TNM (T, tumour size; N, lymph nodes infiltrated by tumour cells; and M, metastasis) classification were not observed.

Our estimation of P-gp at the protein level determined by immunohistochemistry (data not shown) correlated significantly with the *MDR*1 gene expression analysis using cDNA-PCR ($r_s$ = +0.7848, $P < 0.001$, $n$ = 14).

These results were obtained from statistical analysis that tested multiple parameters against each other. However, in breast cancers, the test showed highest correlations exactly for those variables we also found in previous studies in other tumour tissues. Therefore, we suggest that the results might be of importance and should provide a basis for further investigations at the functional level.

Remarkably, expression of the *PKC*$\beta_1$ and $\beta_2$ genes was not detectable either in the primary breast cancer specimens or in material of the MCF7 cell lines by our cDNA-PCR approach applying 32 (*PKC*$\beta_1$) or 28 (*PKC*$\beta_2$) cycles respectively. Therefore, these genes are not listed in the table. Nonetheless, this observation might be important as distinct *PKC*$\beta_1$ and $\beta_2$ gene expression was found in normal lymphocytes under the same conditions (J Beck et al, unpublished results). In fact, in a series of dilution experiments with total RNA prepared from the MCF7 cell line or normal peripheral blood mononuclear cells (PBMCs) we found distinct *PKC*β expression signals if the fraction of added PBMC RNA amounts only to 5%. Thus, a lack of signals corresponding to *PKC*β might indicate that the tumour samples did not contain significant amounts of PBMCs that may give false positives concerning *MDR*1 gene expression.

### DISCUSSION

Standard breast cancer chemotherapeutic regimens include doxorubicin, taxol and vinblastine. Each of these compounds is included in the resistance profile of either *MDR*1 or *MRP* overexpressing cells. The functional involvement of LRP in a cellular resistance towards these drugs remains to be clarified, however. The activity of topoisomerases supposedly affects the sensitivity towards topoisomerase inhibitors such as doxorubicin, but cellular proliferation activity could influence the effects of antiproliferative drugs in general. Retrospective clinical studies showed that breast cancer tissues with high proliferation indices and a low grade of differentiation generally respond better to chemotherapy (Silvestrini and Daidone, 1993). Our present analysis demonstrates that *Topo* IIα and *cyclin A* gene expression levels are significantly lower in G2 than in G3 classified tumours. This corresponds well to the enhanced proliferation activity and lower differentiation grade of G3 tumours than G2 tumours, and the better response of G3 tumours to chemotherapy.

Furthermore, the *MRP* and *LRP* gene expression levels were significantly higher in G2 tumours than in G3 classified tumours.

© Cancer Research Campaign 1998 

This agrees with a previous study on bladder carcinomas that revealed enhanced *MRP* gene expression levels in the higher differentiated G1 and G2 tumours compared with G3 tumours (Clifford et al, 1996). Thus, our results might explain the better responsiveness of G3 staged breast cancers to chemotherapeutic regimens, which may depend not only on the enhanced proliferation rate together with higher activity of *Topo* IIα but also on the lower expression levels of the *MRP* and *LRP* genes compared with G2 staged tumours. In contrast, the drug resistance generally seen in more differentiated primary breast cancers with lower proliferative activity appears to be mediated by multiple factors. However, it is too early to take the observed variations in gene expression levels between the G2/G3 cohorts as prognostic factors for the outcome of treatment in the case of patients included in our study because the observation period after the date of primary diagnosis is still too short.

Recently, we demonstrated in AML blasts a significant positive correlation between the *PKC*η and *MDR*1 or *MRP* gene expression levels, respectively, and in addition between *PKC*θ and *MRP* levels (Beck et al, 1996a). Furthermore, we found enhanced *PKC*η mRNA expression together with higher expression of *MDR*1, *MRP* and *LRP* in ascites cell aspirates from ovarian cancer patients (Beck et al, 1996*b*). Our analysis on breast cancer samples gives very similar results to these earlier studies, i.e. a significant correlation of relative *PKC*η and *MDR*1, *MRP* or *LRP* gene expression levels, respectively. Therefore, we suggest that this particular PKC isozyme might be directly involved in either the expression status of MDR associated genes or the differentiation stage of primary breast cancer tumour cells. This hypothesis might be supported by recent findings that PKCη is predominantly located in the cell nucleus of skin-derived human cells. Accordingly, a possible direct regulatory function of PKCη at the transcriptional level was suggested (Greif et al, 1994).

However, other investigators have already analysed *MDR*1/P-gp expression in breast cancers using different methods. Although these results showed a great variability, the majority of investigators found relatively high *MDR*1/P-gp expression in tumours treated with drugs involved in P-gp-mediated MDR (Goldstein et al, 1989; Schneider et al, 1989; Ro et al, 1990; Sanfilippo et al, 1991). Therefore, it seems interesting to determine whether and to what extent a possible induction of resistance-associated genes in response to chemotherapeutic regimens might be controlled by PKCη.

### ABBREVIATIONS

LRP, lung cancer resistance related protein; MDR, multidrug resistance; MRP, multidrug resistance-associated protein; P-gp, P-glycoprotein; PKC, protein kinase C, Topo II, topoisomerase II.

### ACKNOWLEDGEMENTS

This work was supported by the 'Wilhelm Sander-Stiftung, Neustadt/Donau' and by the 'Fortüne-Programm', University of Tübingen, Germany.

### REFERENCES

Beck J, Handgretinger R, Dopfer R, Klingebiel T, Niethammer D and Gekeler V (1995) Expression of MDR1, MRP, topoisomerase IIα/β, and cyclin A in primary or relapsed states of acute lymphoblastic leukaemias. *Br J Haematol* **89**: 356–363

Beck J, Handgretinger R, Klingebiel T, Dopfer R, Schaich M, Ehninger G, Niethammer D and Gekeler V (1996*a*) Expression of PKC isozyme and MDR-associated genes in primary and relapsed state AML. *Leukemia* **10**: 426–433

Beck J, Regele B, Brügger D, Dietl J, Scheper RJ, Niethammer D, Bader P, Hirsch HA and Gekeler V (1996*b*) Expression of genes (MDR1, MRP, LRP, topoisomerases, PKC isozymes) possibly involved in drug resistance of ovarian carcinoma ascites cell aspirates. *Proc Am Assoc Cancer Res* **37**: 309

Batist G, Tulpule A, Sinha BK, Katki AG, Meyers CE and Cowan KH (1986) Overexpression of a novel anionic glutathione transferase in multidrug-resistant human breast cancer cells. *J Biol Chem* **261**: 15544–15549

Blobe GC, Obeid LM and Hannun YA (1994) Regulation of protein kinase C and role in cancer biology. *Cancer Metast Rev* **13**: 411–431

Bloom HJG and Richardson WW (1957) Histological grading and prognosis in breast cancer. *Br J Cancer* **11**: 359–377

Chambers TC, Chalikonda I and Eilon G (1990*a*) Correlation of protein kinase C translocation, P-glycoprotein phosphorylation and reduced drug accumulation in multidrug resistant human KB cells. *Biochem Biophys Res Commun* **169**: 253–259

Chambers TC, McAvoy EM, Jacobs JW and Eilon G (1990*b*) Protein kinase C phosphorylates P-glycoprotein in multidrug resistant human KB carcinoma cells. *J Biol Chem* **265**: 7679–7686

Chaudhary PM and Roninson IB (1993) Induction of multidrug resistance in human cells by transient exposure to different chemotherapeutic drugs. *J Natl Cancer Inst* **85**: 632–639

Chirgwin JM, Przybyla AE, MacDonald RJ and Rutter WJ (1979) Isolation of biological active ribonucleic acid from sources enriched in ribonuclease. *Biochemistry* **18**: 5294–5299

Clifford SC, Neal DE and Lunec J (1996) Alterations in expression of the multidrug resistance-associated protein (MRP) gene in high-grade transitional cell carcinoma of the bladder. *Br J Cancer* **73**: 659–666

Cordell J, Falini B, Erber W, Ghosh A, Abdulaziz Z, MacDonald S, Pulford K, Stein H and Mayson D (1984) Immunoenzymatic labeling of monoclonal antibodies using immune complexes of alkaline phosphatase and monoclonal anti-alkaline phosphatase (APAAP complexes). *J Histochem Cytochem* **32**: 219–229

Gekeler V, Frese G, Diddens H and Probst H (1988) Expression of a P-glycoprotein gene is inducible in a multidrug-resistant human leukemia cell line. *Biochem Biophys Res Commun* **155**: 754–760

Gekeler V, Beck J, Noller A, Wilisch A, Frese G, Neumann M, Handgretinger R, Ehninger G, Probst H and Niethammer D (1994) Drug-induced changes in the expression of MDR-associated genes: investigations on cultured cell lines and chemotherapeutically treated leukemias. *Ann Hematol* **69**: S19–24

Gekeler V, Boer R, Ise W, Sanders KH, Schächtele C and Beck J (1995) The specific bisindolylmaleimide PKC-inhibitor GF 109203X efficiently modulates MRP-associated multiple drug resistance. *Biochem Biophys Res Commun* **206**: 119–126

Gekeler V, Boer R, Überall F, Ise W, Schubert C, Utz I, Hofmann J, Sanders KH, Schächtele C, Klemm K and Grunicke H (1996) Effects of the selective bisindolylmaleimide protein kinase C inhibitor GF109203X on P-glycoprotein mediated multidrug resistance. *Br J Cancer* **74**: 897–905

Germann UA (1996) P-glycoprotein-a mediator of multidrug resistance in tumour cells. *Eur J Cancer* **32A**: 927–944

Germann UA, Chambers TC, Ambudkar SV, Licht T, Cardarelli CO, Pastan I and Gottesman MM (1996) Characterization of phosphorylation-defective mutants of human P-glycoprotein expressed in mammalian cells. *J Biol Chem* **271**: 1708–1716

Goldstein LJ, Galski H, Fojo A, Willingham M, Lai SL, Gazdar A, Pirker R, Green A, Crist W, Brodeur GM, Lieber M, Cossman J, Gottesman MM and Pastan I (1989) Expression of a multidrug resistance gene in human cancers. *J Natl Cancer Inst* **81**: 116–124

Greif H, Ben-Chaim J, Shimon T, Bechor E, Eldar H and Livneh E (1994) The protein kinase C-related PKC-L(η) gene product is localized in the cell nucleus. *Mol Cell Biol* **12**: 1304–1311

Grunicke H, Hofmann J, Utz I and Überall F (1994) Role of protein kinases in antitumour drug resistance. *Ann Haematol* **69**: 1–6

Izquierdo MA, Scheffer GL, Flens MJ, Schroeijers AB, van der Valk P, Scheper RJ (1996) Major vault protein LRP-related multidrug resistance. *Eur J Cancer* **32A**: 979–984

Loe DW, Deeley RG and Cole SP (1996) Biology of the multidrug resistance-associated protein, MRP. *Eur J Cancer* **32A**: 945–957

Nitiss JL and Beck WT (1996) Antitopoisomerase drug action and resistance. *Eur J Cancer* **32A**: 958–966

Pines J and Hunter T (1990) Human cyclin A is an adenovirus E1A-associated protein p60 and behaves differently from cyclin B. *Nature* **346**: 760–763

© Cancer Research Campaign 1998

Ro J, Sahin A, Ro JY, Fritsche H, Hortobagyi G and Blick M (1990) Immunohistochemical analysis of P-glycoprotein expression correlated with chemotherapy resistance in locally advanced breast cancer. *Hum Pathol* **21**: 787–791

Sanfilippo O, Ronchi E, De Marco C, Di Franzo G and Silvestrini R (1991) Expression of P-glycoprotein in breast cancer tissue and in vitro resistance to doxorubicin and vincristine. *Eur J Cancer* **27**: 155–158

Sato W, Yusa K, Naito M and Tsuruo T (1990) Staurosporine, a potent inhibitor of C-kinase, enhances drug accumulation in multidrug-resistant cells. *Biochem Biophys Res Commun* **173**: 1252–1257

Scala S, Dickstein B, Regis J, Szallasi Z, Blumberg PM, Bates SE, Benjamin C, Osborn L, Lobb R and Harlan JM (1995) Bryostatin 1 affects P-glycoprotein phosphorylation but not function in multidrug-resistant human breast cancer cells. *Clin Cancer Res* **1**: 1581–1587

Schneider J, Bak M, Efferth T, Kaufmann M, Mattern J and Volm M (1989) P-glycoprotein expression in treated and untreated human breast cancer. *Br J Cancer* **60**: 815–818

Silvestrini R and Daidone MG (1993) Review of proliferative variables and their predictive value. In *Recent Results in Cancer Research.* Senn H, Gelber R, Goldhirsch A and Thürlimann B (eds), pp. 71–77. Springer: Heidelberg

Yu G, Ahmad S, Aquino A, Fairchild CR, Trepel JB, Ohno S, Suzuki K, Tsuruo T, Cowan KH and Glazer RI (1991) Transfection with protein kinase C alpha confers increased multidrug resistance to MCF-7 cells expressing P-glycoprotein. *Cancer Commun* **3**: 181–189

© Cancer Research Campaign 1998